# Selenzyme: enzyme selection tool for pathway design

**DOI:** 10.1093/bioinformatics/bty065

**Published:** 2018-02-07

**Authors:** Pablo Carbonell, Jerry Wong, Neil Swainston, Eriko Takano, Nicholas J Turner, Nigel S Scrutton, Douglas B Kell, Rainer Breitling, Jean-Loup Faulon

**Affiliations:** BBSRC/EPSRC Manchester Centre for Synthetic Biology of Fine and Speciality Chemicals (SYNBIOCHEM), Manchester Institute of Biotechnology; BBSRC/EPSRC Manchester Centre for Synthetic Biology of Fine and Speciality Chemicals (SYNBIOCHEM), Manchester Institute of Biotechnology; BBSRC/EPSRC Manchester Centre for Synthetic Biology of Fine and Speciality Chemicals (SYNBIOCHEM), Manchester Institute of Biotechnology; BBSRC/EPSRC Manchester Centre for Synthetic Biology of Fine and Speciality Chemicals (SYNBIOCHEM), Manchester Institute of Biotechnology; School of Chemistry, The University of Manchester, Manchester M1 7DN, UK; BBSRC/EPSRC Manchester Centre for Synthetic Biology of Fine and Speciality Chemicals (SYNBIOCHEM), Manchester Institute of Biotechnology; School of Chemistry, The University of Manchester, Manchester M1 7DN, UK; BBSRC/EPSRC Manchester Centre for Synthetic Biology of Fine and Speciality Chemicals (SYNBIOCHEM), Manchester Institute of Biotechnology; School of Chemistry, The University of Manchester, Manchester M1 7DN, UK; BBSRC/EPSRC Manchester Centre for Synthetic Biology of Fine and Speciality Chemicals (SYNBIOCHEM), Manchester Institute of Biotechnology; School of Chemistry, The University of Manchester, Manchester M1 7DN, UK; BBSRC/EPSRC Manchester Centre for Synthetic Biology of Fine and Speciality Chemicals (SYNBIOCHEM), Manchester Institute of Biotechnology; School of Chemistry, The University of Manchester, Manchester M1 7DN, UK; BBSRC/EPSRC Manchester Centre for Synthetic Biology of Fine and Speciality Chemicals (SYNBIOCHEM), Manchester Institute of Biotechnology; School of Chemistry, The University of Manchester, Manchester M1 7DN, UK; MICALIS, INRA-AgroParisTech, Domaine de Vilvert, 78352 Jouy en Josas Cedex, France

## Abstract

**Summary:**

Synthetic biology applies the principles of engineering to biology in order to create biological functionalities not seen before in nature. One of the most exciting applications of synthetic biology is the design of new organisms with the ability to produce valuable chemicals including pharmaceuticals and biomaterials in a greener; sustainable fashion. Selecting the right enzymes to catalyze each reaction step in order to produce a desired target compound is, however, not trivial. Here, we present Selenzyme, a free online enzyme selection tool for metabolic pathway design. The user is guided through several decision steps in order to shortlist the best candidates for a given pathway step. The tool graphically presents key information about enzymes based on existing databases and tools such as: similarity of sequences and of catalyzed reactions; phylogenetic distance between source organism and intended host species; multiple alignment highlighting conserved regions, predicted catalytic site, and active regions and relevant properties such as predicted solubility and transmembrane regions. Selenzyme provides bespoke sequence selection for automated workflows in biofoundries.

**Availability and implementation:**

The tool is integrated as part of the pathway design stage into the design-build-test-learn SYNBIOCHEM pipeline. The Selenzyme web server is available at http://selenzyme.synbiochem.co.uk.

**Supplementary information:**

Supplementary data are available at *Bioinformatics* online.

## 1 Introduction

In recent years, powerful bioinformatics tools are being increasingly integrated into systems and synthetic biology pipelines (Carbonell *et al.*, 2016). Synthetic biology employs the engineering principle of an iterative Design–Build–Test–Learn cycle. In the case of developing engineered organisms for the production of high-value compounds, the Design stage involves the identification of the most suitable combinations of starting substrates, enzymes, regulatory components and chassis organism for the desired biosynthetic pathway. Hence, bioinformatics tools used at this stage usually carry out database mining in order to search for the best candidate parts and devices. Some of these tools are capable of establishing possible pathways leading to a target compound by using sets of encoded reaction rules, like RDM patterns in PathPred (Moriya *et al.*, 2010); Bond-Electron matrices in BNICE (Hadadi *et al.*, 2016) or reaction SMARTS in RetroPath 2.0 (Delépine *et al.*, 2018). In order to select candidate sequences for enzymes at each step of the identified pathways, several tools provide different solutions, including antiSMASH for biosynthetic gene clusters (Weber *et al.*, 2015), as well as tools based on reaction homologies like EC-Blast (Rahman *et al.*, 2014) or machine learning (Mellor *et al.*, 2016). Here, we extend such capabilities through Selenzyme, sequence selection with the ability to mine SMARTS reaction rules. The tool is part of the SYNBIOCHEM automated Design/Build/Test/Learn pipeline for microbial fine chemical production, which integrates computational, robotics, assembly, analytics and machine learning platforms. Selenzyme is fed from the output of the pathway discovery workflow RetroPath 2.0 (Delépine *et al.*, 2018) and will be integrated with the downstream tool for part optimization PartsGenie (Swainston *et al.*, 2017b).

## 2 Design and implementation

Our goal is to mine for candidate enzyme sequences for any desired target reaction or set of reactions in a pathway. A unique feature of Selenzyme is that target reactions do not necessarily need to exist in databases, enabling the tool to search for alternative routes for biosynthesis, degradation and transport either for natural or non-natural products. Selenzyme uses SYNBIOCHEM’s biochem4j graph database (Swainston *et al.*, 2017a) (June 2017) as its main data source. The database contains relevant information on relationships between reactions (36765), chemicals (19735), enzymes (245704) and organisms (8431).

A Selenzyme query consists of a target reaction, represented in .rxn format (MDL), a character string using either the SMARTS format or the derivative SMIRKS representation for reaction rules (http://www.daylight.com/), an external database id or an EC number. The query is then screened against the reaction database in order to look for similar chemical transformations. Individual Tanimoto similarities between chemicals are obtained using pre-selected fingerprints (http://www.rdkit.org). The efficiency of such similarity screening method was previously validated for the set of reaction rules used by the RetroPath 2.0 workflow (Delépine *et al.*, 2018). The user can choose to rank similarities in both reaction directions or to use only the direction of the reaction based on a consensus list that has been generated according to reaction information based on curated information from MetaCyc (Supplementary Material). The algorithm proceeds through the list of reactions ranked by decreasing similarity and collects annotated sequences associated with the reactions. For each sequence, several useful properties such as phylogenetic distance to the host chassis and predicted physicochemical properties computed via the EMBOSS suite are collected. Optionally, a multiple sequence alignment (MSA) can be generated using T-Coffee (Taly *et al.*, 2011) and visualized using MSAviewer (Yachdav *et al.*, 2016). The output generates a .csv file containing the list of top sequence candidates.

On top of this core tool, there is a web server built using Flask in Python. Once the reaction query is submitted, the ranked list of sequence candidates is presented as an interactive table, which can be sorted on user-defined summary scores based on a weighted average of selected columns or properties. Additional sequences can be added or removed from the table. Moreover, a RESTful service has been implemented, so that Selenzyme can accept multiple queries from any other web-based application. An example KNIME node (O’Hagan and Kell, 2015) is available so that the reaction query can be generated from chemoinformatics operations within a workflow (Supplementary Material).

**Fig. 1.**
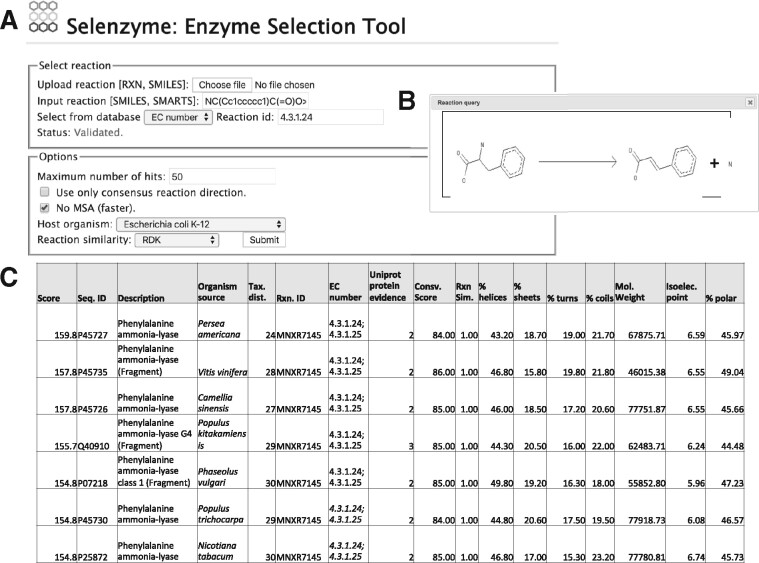
Query submission to Selenzyme through the web interface and resulting table of top ranked sequences on the default score with associated properties and cross-references to databases. (**A**) Only reactions in the preferred direction in the database were considered for the (**B**) target reaction. (**C**) The results page provides links for downloading the table, or the multiple sequence alignment and links to external databases

Selenzyme is a flexible tool that can provide enzyme selection solutions in different scenarios. We consider for instance the biosynthetic pathway of the flavonoid pinocembrin engineered in the chassis organism *Escherichia coli*. The pathway consists of four reaction steps (PAL, 4CL, CHS and CHI). The reactions were defined using a molecule editor and exported as SMILES strings. Figure 1 shows the top selected sequences for PAL based on the pre-defined combined score of reaction similarity, phylogenetic distance, UniProt protein evidence and sequence conservation.

## Funding

This work was supported by BBSRC/EPSRC [grant number BB/M017702/1], ‘Centre for synthetic biology of fine and speciality chemicals (SYNBIOCHEM)’ and ANR [grant number ANR-15-CE1-0008].

*Conflict of Interest*: none declared.

## Supplementary Material

Supplementary Data
