# Supplementary material for: Selenzyme: enzyme selection tool for pathway design
Source: Bioinformatics. 2018 Feb 7;34(12):2153–4. doi: 10.1093/bioinformatics/bty065 (PMC9881682; doi:10.1093/bioinformatics/bty065)
Supplement: Supplementary Data [file bioinformatics_34_12_2153_s2.pdf]

Supplementary data

A.1 Computing consensus direction for reactions

A.2 Querying Selenzyme for reaction and pathway sequence selection using KNIME workflows

A.3 Querying Selenzyme with a generalized reaction rule

A.4 Querying Selenzyme from RetroPath2.0 scope viewer

A.5 Additional references

A.1 Computing consensus direction for reactions

Reaction databases are often annotated in the less favorable direction (under physiological conditions). In order to determine the most plausible direction for a reaction when a preferred direction exists, some heuristics were applied based on curated information from MetaCyc and cofactor exchange pattern frequencies according to the EC number.

The algorithm is as follows:

- **A.** Initial curated information about reaction direction was collected from MetaCyc.
- **B.** Initial curated information about main reactants and cofactors for each reaction was collected from MetaCyc pathway information when available.
- **C.** A list of currency metabolites was extracted based on frequency of occurrence in Metacyc and biochem4j and manually curated.
- **D.** FOR each reaction in biochem4j:
    - a. Identify left and right cofactors and main reactants based on information from **A**, **B**, **C**.
- **E.** FOR each EC number in biochem4j:
    - a. FOR each reaction annotated with each EC:
        - i. Store frequency of left/right cofactor pairs from **A**, **B**, **C**.
- **F.** FOR each reaction in biochem4j:
    - a. IF direction information from MetaCyc available:
        - i. SET MetaCyc direction from **B**.
    - b. ELSE
        - i. IF reaction has annotated EC number and left/right cofactor pair in *E* for annotated EC:
            1. SET direction with highest frequency from **E**.

A.2 Querying Selenzyme for reaction and pathway sequence selection using KNIME workflows

This example case shows the use of KNIME workflows to interface with Selenzyme. Figure A1 shows an example of enzyme sequence selection for two types of queries: a target reaction and a pathway involving multiple reactions. The workflows are available at http://www.myexperiment.org/packs/734.

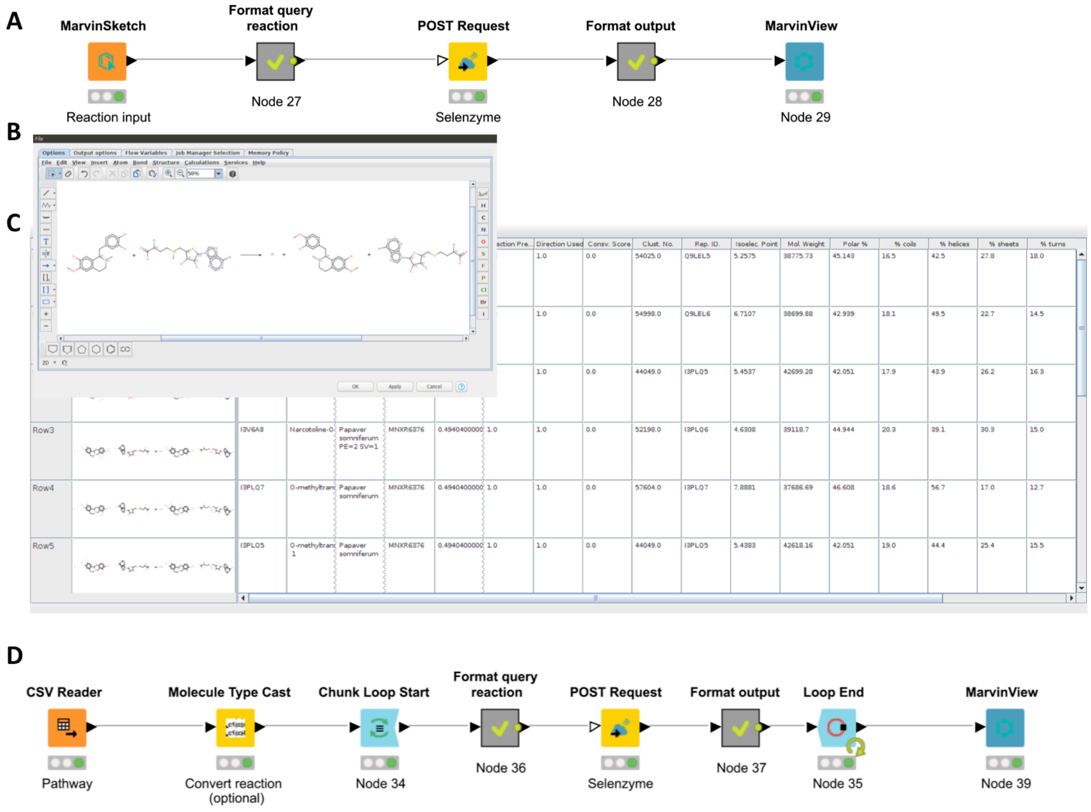

Figure A1. Example of application of the Selenzyme node in KNIME for reaction and pathway enyme selection. (**A**) The reaction is submitted to Selenzyme as a SMARTS query through the RESTful service at http://selenzyme.synbiochem.co.uk. (**B**) The query reaction is sketched using a chemical editor. (**C**) The output of the workflow provides a table containing the top selected sequences and information about their properties. (**D**) Another application of Selenzyme is for querying a collection of reactions, typically a multi-step pathway that can be iteratively queried through a loop.

A.3 Querying Selenzyme with a generalized reaction rule

An application example of querying for enzyme sequences from a generalized reaction rule. We take as an example the first reaction rule from the set of reaction operators that was used in Yim *et al.*, 2011. The associated diagram is shown in Figure A2 and an example output table in Table A1.

Operator class: 1.1.1.-

Description: Oxidoreductase (alcohol to ketone)

Reaction SMARTS:

([#8:4](-[#1])-[#6:2](-[#6,#1:1])(-[#1])-[*,#1:3])>>([*:1]-[#6:2](-[*:3])=[O:4])

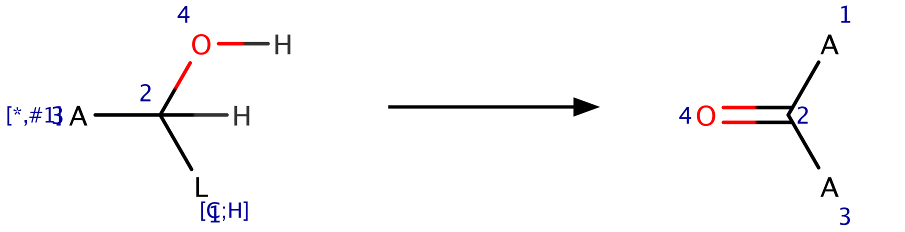

Figure A2. Diagram corresponding to the reaction SMARTS of the operator class 1.1.1.-. The diagram was generated using MarvinView (ChemAxon Ltd.).

Score	Seq. ID	Description	Organism Source	Tax. distance	Rxn. ID	EC Number	Consv. Score	Rxn Sim.
121.536	B1LIP1	S-(hydroxymethyl)glutathione dehydrogenase	Escherichia coli (strain SMS-3-5 / SECEC)	3	MNXR8926	1.1.1.1	50	0.74536
121.536	A7ZX04	S-(hydroxymethyl)glutathione dehydrogenase	Escherichia coli O9:H4 (strain HS)	3	MNXR8926	1.1.1.1	50	0.74536
121.536	Q0TKS7	S-(hydroxymethyl)glutathione dehydrogenase	Escherichia coli O6:K15:H31 (strain 536 / UPEC)	3	MNXR8926	1.1.1.1	50	0.74536
113.536	P39450	S-(hydroxymethyl)glutathione dehydrogenase	Photobacterium damsela subsp. piscicida	11	MNXR8926	1.1.1.1	50	0.74536
98.536	P81431	Alcohol dehydrogenase class-3	Octopus vulgaris PE=1 SV=1	26	MNXR8926	1.1.1.1	50	0.74536
94.536	Q99W07	Alcohol dehydrogenase	Staphylococcus aureus (strain Mu50 / ATCC 700699)	17	MNXR8926	1.1.1.1	37	0.74536
91.536	P14675	Alcohol dehydrogenase 3	Solanum tuberosum	30	MNXR8926	1.1.1.1	47	0.74536
91.536	P9WQC2	Probable alcohol dehydrogenase adh	Mycobacterium tuberculosis (strain CDC 1551 / Oshkosh)	17	MNXR8926	1.1.1.1	34	0.74536
90.536	P0DJA2	Alcohol dehydrogenase 2	Zymomonas mobilis subsp. mobilis (strain ATCC 31821 / ZM4 / CP4)	14	MNXR8926	1.1.1.1	30	0.74536
90.536	Q6XQ67	Alcohol dehydrogenase 5	Saccharomyces pastorianus	21	MNXR8926	1.1.1.1	37	0.74536
90.536	G5EGA6	Dehydrogenase/reductase SDR family member 4	Caenorhabditis elegans	24	MNXR7089	1.1.1.184	40	0.74536
89.536	Q8VZ49	Alcohol dehydrogenase-like 4	Arabidopsis thaliana	29	MNXR8926	1.1.1.1	44	0.74536
89.536	Q0V7W6	Alcohol dehydrogenase-like 5	Arabidopsis thaliana	29	MNXR8926	1.1.1.1	44	0.74536
89.536	Q75ZX4	Alcohol dehydrogenase 1	Oryza sativa subsp. indica	31	MNXR8926	1.1.1.1	46	0.74536
88.536	P74308	Aldo/keto reductase slr0942	Synechocystis sp. (strain PCC 6803 / Kazusa)	16	MNXR7089	1.1.1.184	30	0.74536
88.536	Q03505	Alcohol dehydrogenase 1	Oryctolagus cuniculus	35	MNXR8926	1.1.1.1	49	0.74536
87.536	P41747	Alcohol dehydrogenase 1	Aspergillus flavus (strain ATCC 200026 / FGSC A1120 / NRRL 3357 / JCM 12722 / SRRC 167)	24	MNXR8926	1.1.1.1	37	0.74536
86.536	Q28960	Carbonyl reductase [NADPH] 1	Sus scrofa	35	MNXR7089	1.1.1.184	47	0.74536
85.536	Q8WNV7	Dehydrogenase/reductase SDR family member 4	Sus scrofa	35	MNXR7089	1.1.1.184	46	0.74536
85.536	Q64413	Alcohol dehydrogenase 1	Geomys bursarius	36	MNXR8926	1.1.1.1	47	0.74536

Table A1. Top sequence candidates in results table for the generalized reaction operator of EC 1.1.1.- (detail).

A.4 Querying Selenzyme from RetroPath2.0 scope viewer

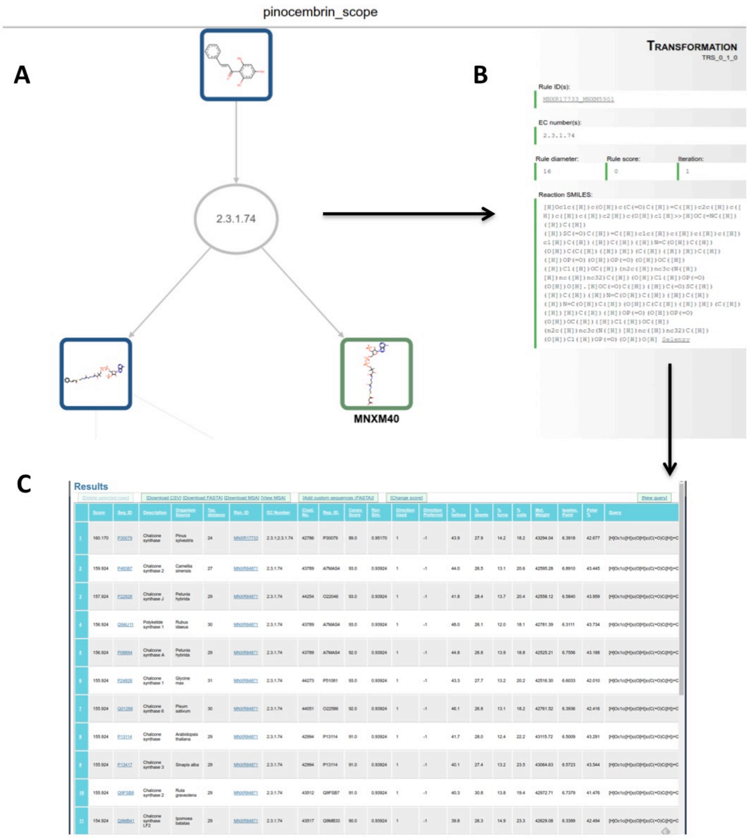

Figure A3. Retrieving sequence selection information for individual steps in the metabolic scope from *Escherichia coli* to pinocembrin in RetroPath2.0. (**A**) An reaction step is zoomed in the viewer. (**B**) Clicking in the enzyme provides information and a link to Selenzyme. (**C**) The link generates a table of selected sequences for the reaction step.

RetroPath2.0 (Delepine et al., 2017) computes the metabolic scope, i.e., the set of heterologous reactions connecting the chassis with the target, between a chassis organism and target compounds. A simple modification of the RetroPath2.0 scope viewer (Figure A3) by adding a link to the Selenzyme RESTful service that queries the SMARTS string of the reaction can be used for further investigation of each catalytic step and selection of candidate sequences.

A.5 Additional references

Yim, H. *et al.* (2011) Metabolic engineering of *Escherichia coli* for direct production of 1,4-butanediol. *Nat. Chem. Biol.*, **7**, 445–452.
